# Photoluminescent coordination polymer bulk glasses and laser-induced crystallization†

**DOI:** 10.1039/d1sc06751f

**Published:** 2022-02-24

**Authors:** Zeyu Fan, Chinmoy Das, Aude Demessence, Ruilin Zheng, Setsuhisa Tanabe, Yong-Sheng Wei, Satoshi Horike

**Affiliations:** AIST-Kyoto University Chemical Energy Materials Open Innovation Laboratory (ChEM-OIL), National Institute of Advanced Industrial Science and Technology (AIST) Yoshida-Honmachi, Sakyo-ku Kyoto 606-8501 Japan horike@icems.kyoto-u.ac.jp; Department of Synthetic Chemistry and Biological Chemistry, Graduate School of Engineering, Kyoto University Katsura, Nishikyo-ku Kyoto 615-8510 Japan; Univ Lyon, Claude Bernard Lyon 1 University, UMR CNRS 5256, Institute of Researches on Catalysis and Environment of Lyon (IRCELYON) Villeurbanne France; Graduate School of Human and Environmental Studies, Kyoto University Kyoto 606-8501 Japan; Institute for Integrated Cell-Material Sciences, Institute for Advanced Study, Kyoto University Yoshida-Honmachi, Sakyo-ku Kyoto 606-8501 Japan; Department of Materials Science and Engineering, School of Molecular Science and Engineering, Vidyasirimedhi Institute of Science and Technology Rayong 21210 Thailand

## Abstract

We synthesized luminescent coordination polymer glasses composed of d^10^ metal cyanides and triphenylphosphine through melt-quenching and mechanical milling protocols. Synchrotron X-ray total scattering measurements and solid-state NMR revealed their one-dimensional chain structures and high structural dynamics. Thermodynamic and photoluminescence properties were tunable by the combination of heterometallic ions (Ag^+^, Au^+^, and Cu^+^) in the structures. The glasses are moldable and thermally stable, and over centimeter-sized glass monoliths were fabricated by the hot-press technique. They showed high transparency over 80% from the visible to near-infrared region and strong green emission at room temperature. Furthermore, the glass-to-crystal transformation was demonstrated by laser irradiation through the photothermal effect of the glasses.

## Abstract 2

Over centimeter-sized luminescent coordination polymer glasses were fabricated. They showed high transparency (over 80%) and strong green emission at room temperature. The glass-to-crystal transformation by laser irradiation was demonstrated.

## Graphical abstract

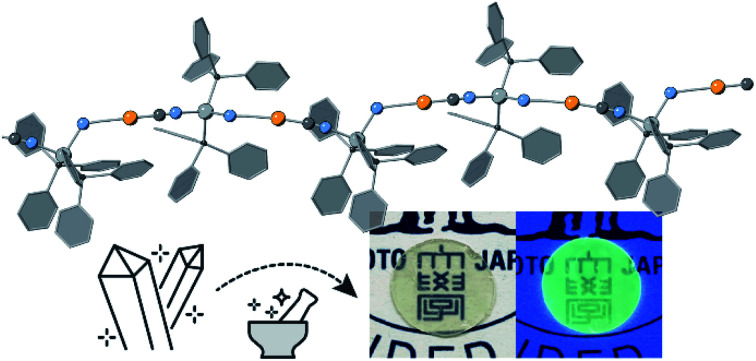

## Introduction

Glassy coordination polymers (CPs) and metal–organic frameworks (MOFs) have been of interest as a new class of amorphous materials.^1–4^ Their structure and properties are distinct from conventional glasses and are controlled by the combination of metal ions and bridging molecular ligands. In addition, the non-crystalline, high processability, and adhesive characteristics have gained research interest in both fundamental behaviors as well as applications such as ion conductive solid electrolytes, gas separation membranes, and electrochemical catalysis.^5–10^

One of the most successful applications of glass, in general, is optics. The transparency and moldability of optically active glass materials have enabled many optical functionalities, including photoconductivity, photon up-conversion, and spectral concentration.^11–13^ However, studies on the optical properties and materials fabrication of CP/MOF glasses are still in their infancy. This is because limited compounds have been found which possess essential criteria for optical materials: (i) strong luminescence, (ii) moldability in bulk size (over a centimeter), and (iii) high transparency over 80%.^14^ The preparation of bulk ZIF-62 glass monoliths with a high transparency up to 90% in the visible and near-infrared region has been reported in a previous report.^15^ Mid-infrared luminescence was achieved through Co^2+^ doping into ZIF-62 glass.^16,17^ Au^+^ and thiophenolate CP only showed red luminescence at low-temperature (−180 °C) and a transparency of 26% at 850 nm.^18^ It is demanded to explore the new luminescent CP/MOF glasses to meet these criteria.

One ligand system to construct CP/MOFs with tunable compositions and dimensionalities is cyanide.^19–21^ CP/MOFs composed of cyanide and dicyanamide are reported to show melting and vitrification behaviors. The flexible coordination bond between cyanides and monovalent d^10^ metal ions is suitable for glass formation.^22–25^ Here we report three cyanide-based CP glasses with one-dimensional (1D) structures {[M^N^(PPh_3_)_2_][M^C^(CN)_2_]}_*n*_ (M^N^, M^C^ = Cu^+^, Ag^+^ or Au^+^).^26^ The glassy state was obtained from crystalline states through either the melt-quenching or ball milling process. The alternative monovalent metal centers adjust the thermodynamic and photoluminescence properties of glasses. The CP glasses formed a centimeter-scale transparent monolith by a simple vacuum hot-press technique and showed strong green emission at room temperature. Moreover, the laser triggered phase transition from glass to the crystal in the monolith was demonstrated.

## Results and discussion

### Crystal structures

**Fig. 1:**
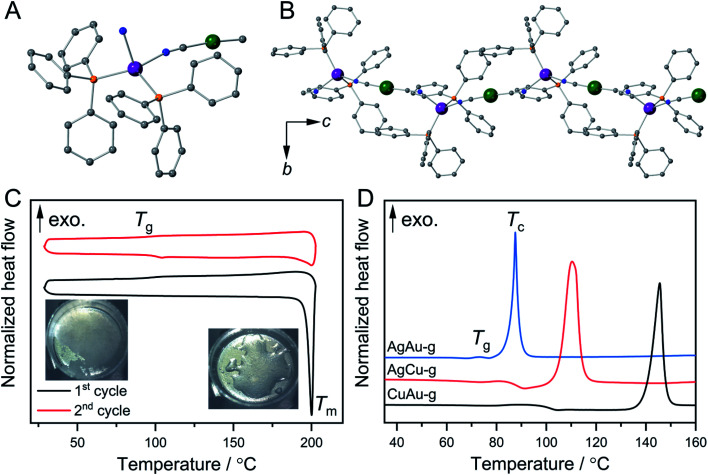
Crystal structures of AgAu (A) in the Ag^+^ coordination sphere and (B) 1D chains along the *c*-axis. Grey, blue, orange, violet, and green are C, N, P, Ag, and Au, respectively. (C) First (black) and second (red) DSC upscan/downscan cycles of CuAu. Melting temperature *T*_m_ and glass transition temperature *T*_g_ are shown. (D) First DSC upscan of M^N^M^C^-g from 35 to 160 °C. The heating rate is 10 °C min^−1^.

**Scheme 1:**
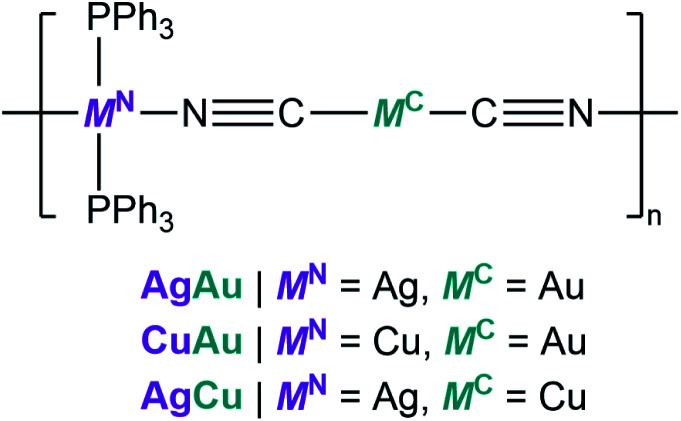
Illustration of the chemical structures of M^N^M^C^.

Fig. 1A and B show the crystal structures of {[Ag(PPh_3_)_2_][Au(CN)_2_]}_*n*_ (AgAu).^27^ Ag^+^ has a tetrahedral configuration with two phosphine atoms from triphenylphosphine (PPh_3_) and two nitrogen atoms from cyanides. These tetrahedra are linked to Au^+^ to give 1D zig-zag chains along the *c*-axis. Weak interactions including Au⋯H and C–H⋯π interactions exist between the 1D chains. Guest-containing {[Ag(PPh_3_)_2_][Cu(CN)_2_]}_*n*_·*x*H_2_O and {[Cu(PPh_3_)_2_][Au(CN)_2_]}_*n*_·*x*H_2_O were synthesized as microcrystalline powders following the reported procedures (see the ESI†).^26–28^ Their guest-free phases, {[Ag(PPh_3_)_2_][Cu(CN)_2_]}_*n*_ (CuAg) and {[Cu(PPh_3_)_2_][Au(CN)_2_]}_*n*_ (CuAu), were obtained by removing the guest water molecules upon heating. Powder X-ray diffraction (PXRD) patterns of AgCu and CuAu are similar to that of AgAu, indicating that they have the same 1D chain structures (Fig. S1A, S2A and S3A†). We conducted Rietveld refinement analysis of AgCu and CuAu using synchrotron PXRD data at 25 °C. These two crystal structures have the same space group as AgAu, and the structures are identical with slight differences of cell parameters (Fig. S4†). Elemental analyses and FT-IR spectra also confirmed the compositions and same structures for the three compounds. The compounds are hereafter denoted as M^N^M^C^, where M^C^ is the metal center that links to the C atoms, and M^N^ is the metal center that links to the N atoms (Scheme 1).

### Crystal melting and formation of melt-quenched glass (MQG)

Thermogravimetric analysis (TGA) was carried out to check the thermal behaviors of M^N^M^C^. AgCu and CuAu showed no weight loss at 200 °C, while AgAu exhibited higher thermal stability and no weight loss at 220 °C. All M^N^M^C^s melt upon heating. Differential scanning calorimetry (DSC) clarified that the melting temperatures (*T*_m_) of AgAu, AgCu, and CuAu are 218, 198, and 197 °C, respectively. The melt phases of CuAu and AgAu are colorless, and that of AgCu is yellow (Fig. S1B, S2B and S3B†). The enthalpy and entropy changes in phase transition (Δ*H*_m_ and Δ*S*_m_) calculated from DSC are summarized in Table S1.†AgAu exhibits higher *T*_m_ and Δ*H*_m_ (65 kJ mol^−1^) than CuAu and AgCu, suggesting the stronger coordination bonds and M^C^⋯H and C–H⋯π interactions. Glass transition (*T*_g_) of CuAu was observed at 100 °C in the first cooling process, and the second heating–cooling cycle (Fig. 1C). We denote the glassy state of CuAu obtained by melt-quenching as CuAu-MQG hereafter. Glassy materials usually exhibit a *T*_g_/*T*_m_ (K/K) value of 2/3, which is summarized as the empirical Kauzmann 2/3 rule.^29,30^CuAu-MQG has a *T*_g_/*T*_m_ value of 0.79, which is higher than the commonly observed 2/3 ratio (=0.67), indicative of its high glass forming ability.^31^AgAu and AgCu did not form glass but crystallized upon cooling as confirmed by DSC and PXRD (Fig. S5†). Although these compounds have identical crystal structures, they show different *T*_m_ and Δ*H*_m_. Higher Δ*H*_m_ in AgAu and AgCu than in CuAu suggests the stronger interactions (coordination bonds and other non-covalent interactions) in coordination polymers, which result in faster recrystallization kinetics than glass formation.^32^ Elemental analysis suggested that the composition of M^N^M^C^s does not change by the melt-quench process (Table S2†).

### Formation of mechanically induced glasses (MIGs) and thermal properties

Ball milling of CP crystals is another procedure to obtain a glassy state.^5,33^ Ball milling of three M^N^M^C^s under an Ar atmosphere yielded the MIGs that are denoted as M^N^M^C^-g hereafter. M^N^M^C^-g exhibited broad PXRD patterns suggesting their amorphous nature. TGA confirmed that the decomposition temperatures of M^N^M^C^-g are the same as M^N^M^C^ (Fig. S1–S3†). DSC confirmed that the onset *T*_g_ of M^N^M^C^-g are 73 °C, 84 °C, and 100 °C for AgAu-g, AgCu-g, and CuAu-g, respectively. Above *T*_g_, we found the exothermal peaks which correspond to crystallization. The crystallization peak temperatures (*T*_c_) are 88 °C, 116 °C, and 146 °C for AgAu-g, AgCu-g, and CuAu-g (Fig. 1D). PXRD confirmed that all M^N^M^C^-g crystallized back to the original crystal structures M^N^M^C^ (Fig. S1A, S2A and S3A†). Different thermal behaviors were found between CuAu-MQG and CuAu-g in the heating process of the DSC profile, in which CuAu-MQG did not show *T*_c_ at 146 °C. This is due to their different particle sizes and surface areas. The larger surface area of CuAu-g induces higher probability for crystallization.^23^ We ground CuAu-MQG into a powder and then performed DSC. *T*_g_ and *T*_c_ at 100 and 146 °C were observed in the first heating process of the DSC profile, which is identical to CuAu-g. No exothermal peak was observed in the second heating process because of the formation of the bulk melt-quenched glass (Fig. S6†). CuAu-g exhibited the largest temperature window between *T*_g_ and *T*_c_ of 46 °C among M^N^M^C^-g (15 °C for AgAu-g and 32 °C for AgCu-g). The large operating window of CuAu-g provides an opportunity for glass engineering since crystallization hardly happens during the thermal treatment.^34^

### Structural characterization of MIGs

FT-IR and Raman spectra were measured to study the local structural differences between M^N^M^C^ and M^N^M^C^-g, as the –C≡N bond (νCN) vibration is sensitive to its chemical environment.^35^ The four cyanide vibration peaks in CuAu are due to the coupling of stretching modes between cyanide bonds.^23,36^ In CuAu-g, these vibrational peaks were broader and red-shifted to 2158 and 2118 cm^−1^, indicating the elongation of the M^N^–N coordination bond (Fig. S7†).^37,38^ Only the broadness of the νCN bands is observed in AgCu-g and AgAu-g with a negligible shift (Fig. S8 and S9†). In the Raman spectra of CuAu, the peak at 128.9 cm^−1^ that is assigned to Cu–P vibration disappeared by vitrification.^39,40^ Same as CuAu-g, the peaks corresponding to Ag–P at 125.1 and 126.7 cm^−1^ in AgCu-g and AgAu-g disappeared (Fig. S10–S12†). The loss of crystallinity results in a broadening or disappearance of peaks in the Raman spectra.^41–43^

**Fig. 2:**
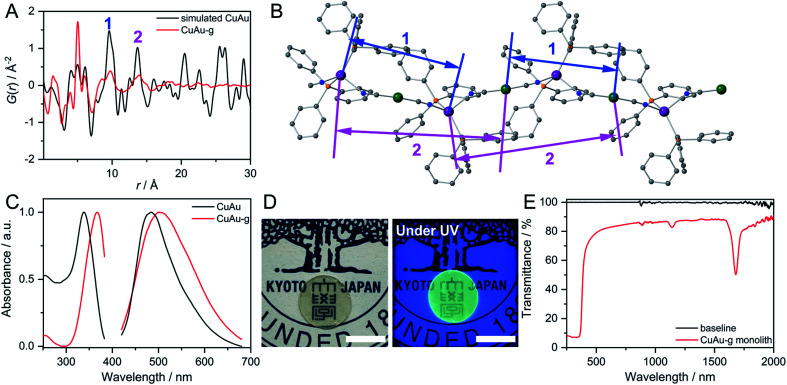
(A) PDF profiles of simulated CuAu and CuAu-g at 25 °C. (B) Atomic pairs corresponding to peak 1 and 2 in the PDF profile of CuAu. Grey, blue, orange, violet, and green are C, N, P, Cu, and Au, respectively. (C) Excitation and emission spectra of CuAu and CuAu-g at 25 °C. (D) Transparent monolith of CuAu-g made by hot-pressing with/without UV light. Scale bar is 1 cm. (E) Transmittance of the CuAu-g monolith from 250 nm to 2000 nm.

We performed X-ray absorption spectroscopy (XAS) for CuAu and CuAu-g to understand the local structure around Cu^+^. They showed identical X-ray absorption near-edge structure (XANES) spectra with the intense rising-edge 1s to 4p transition lies at 8980 eV, which is identical to Cu_2_O. This suggests that Cu in CuAu-g maintains the +1 oxidation state (Fig. S13†).^44^ The radial distribution functions (RDFs), obtained from the Fourier-transformed Cu K-edge extended X-ray absorption fine structure (EXAFS) spectra, showed a similar shape in the range of 1.2 to 2.3 Å corresponding to the first coordination sphere of Cu^+^. The lower peak intensity in CuAu-g than in CuAu is due to the structural disorder. The fitting results of *k*^3^ weighted RDFs indicate that the coordination number of Cu^+^ is 4.3 ± 0.6 and 4.2 ± 1.0 in CuAu and CuAu-g (Fig. S14†). The results suggest that the tetrahedral coordination geometry of Cu is preserved in the glassy state. X-ray total scattering measurements and corresponding pair distribution function (PDF) analyses were carried out to investigate the structural periodicity in M^N^M^C^-g. We compared the PDF profile of M^N^M^C^-g with the simulated profile of M^N^M^C^. The peaks at 5.0, 9.8 (peak 1), and 13.6 Å (peak 2) in Fig. 2A of CuAu-g correspond to Cu–NC–Au, Cu–Au(CN)_2_–Cu/Au–Cu(NC)_2_–Au, and Cu–Au(CN)_2_–Cu–NC–Au (Fig. 2A and B). This indicates the preservation of the periodic order along the 1D chain in the glassy state. PDF profiles of AgCu-g and AgAu-g showed the same periodic features as CuAu-g (Fig. S15†). We conclude that the 1D chain structures are mainly preserved in M^N^M^C^-g with disordered packing modes.

Solid-state NMR (SSNMR) at 25 °C was carried out to study the structural dynamics (Fig. S16†). ^31^P NMR spectra of CuAu exhibited peaks at 8.1, 3.2, −1.6, −6.6, and −12.4 ppm ascribed to Cu–P and P–P couplings. Broad peaks at −2.5 and 40 ppm were observed in CuAu-g due to the structural disorder. The peak at 40 ppm is ascribed to Cu(PPh_3_)_2_NC units.^45^ The ^1^H spin-lattice relaxation time (*T*_1_) of PPh_3_ in CuAu-g is 2.60 s, significantly shorter than that of CuAu (47.37 s). As a same trend, *T*_1_ values of ^1^H for AgCu-g and AgCu are 3.03 and 121.21 s, respectively. The shorter *T*_1_ in M^N^M^C^-g suggests the higher mobility of PPh_3_ ligands by the smaller M^C^⋯H and C–H⋯π interactions as a result of weakened inter-chain interaction.^46^

### Photoluminescence properties of MIGs

The excitation and emission spectra of CuAu and CuAu-g are shown in Fig. 2C. CuAu showed the emission peak at 484 nm and excitation maximum at 338 nm. Previous studies have confirmed that the emission of M^N^M^C^ is from metal-to-metal-to-ligand charge transfer (MMLCT).^28^ The LUMO of M^N^M^C^ is composed of π*(C≡N) and p*(M^N^) orbitals and HOMO is composed of the *d*_*xy*^2^_ orbital of the M^N^N_2_P_2_ chromophore with σ(M^N^–P) bonding, π-orbital from phenyl rings and π(C≡N) bonding. The excitation and emission maxima red-shifted to 367 and 500 nm in CuAu-g. The emission and excitation spectra of CuAu-MQG show the same feature as CuAu-g (Fig. S17A†). The alteration of coordination bond lengths and molecular packing upon glass formation would affect the electronic structures and induce different photophysical properties. The changes contribute to the different energy levels of bonding orbitals and antibonding orbitals. This causes the smaller HOMO–LUMO bandgap and the bathochromic shift of the MMLCT emission band in CuAu-g.^47,48^ The full width at half maximum of the emission spectra of CuAu and CuAu-g is 96 and 140 nm. The larger FWHM is consistent with its disordered structure. AgCu, AgAu, and their glassy states exhibit weaker emissions than CuAu and CuAu-g. In AgCu and AgAu, the emission band did not significantly shift by amorphization because the HOMO–LUMO band gap is kept at a similar level (Fig. S17B and C†). M^N^M^C^ exhibited two-component decay of the photoluminescence lifetime in the microsecond time scale, implying the phosphorescence mechanism. CuAu has 22.7 μs (61.7%) and 4.6 μs (38.3%) of photoluminescence lifetimes. CuAu-g exhibits 8.0 μs (36.2%) and 1.4 μs (63.8%). The average photoluminescence lifetime is 15.8 μs for CuAu and 3.8 μs for CuAu-g. The shorter lifetime in a glassy state than that of the crystal is due to the following reasons. (i) Increased energy trapping states by the formation of defects and (ii) enhanced molecular dynamics in glassy states as confirmed by the relaxation time of SSNMR.^49,50^ The other two glasses M^N^M^C^-g also showed a shorter photoluminescence lifetime than M^N^M^C^ (Fig. S18†).

### Preparation of a transparent bulk glass monolith from MIGs and laser-induced crystallization

One of most important advantages of glass is the processability of materials in a wide range of scales. We prepared a bulk glass monolith by the vacuum hot-press technique. We first preheated the pellet of CuAu-g at 105 °C which is higher than its *T*_g_ (100 °C). The pressure of 60 kN was then applied to the pellet and maintained for 30 min. A bulk glass monolith with a diameter of 1.273 cm and thickness of 0.438 mm was obtained after cooling to 25 °C. The prepared CuAu-g monolith maintains its amorphous state as confirmed by PXRD (Fig. S19A†). It exhibits high transparency and maintains green emission under UV light irradiation (365 nm, Fig. 2D). The transmittance is over 80% from 400 to 2000 nm, comparable to soda-lime glass which is used for windows.^51–53^ The absorption below 400 nm is ascribed to the MMLCT absorption band. Two absorption peaks observed at 1139 and 1680 nm are from the C–H stretching from the phenyl groups of PPh_3_, as these two peaks are observed at similar wavelengths from the reflectance diffusion spectra of PPh_3_ (Fig. 2E and S19B†).^54^ SEM and microscope images indicate that the monolith has a crack-free and smooth surface (Fig. S20A–C†). We prepared the crystalline CuAu pellet under the same hot-press conditions as the control experiment. The obtained crystalline pellet was opaque, and particles separated by grain-boundaries are observed during the SEM of its surface and cross section (Fig. S20D and E†).

A similar procedure was used to prepare the transparent monolith of AgCu-g, and the transmittance is 80% from 400 to 2000 nm (Fig. S21†). On the other hand, the transparent monolith of AgAu-g was not successfully prepared because crystallization occurred during hot-pressing due to its small *T*_g_–*T*_c_ window of 15 °C.

**Fig. 3:**
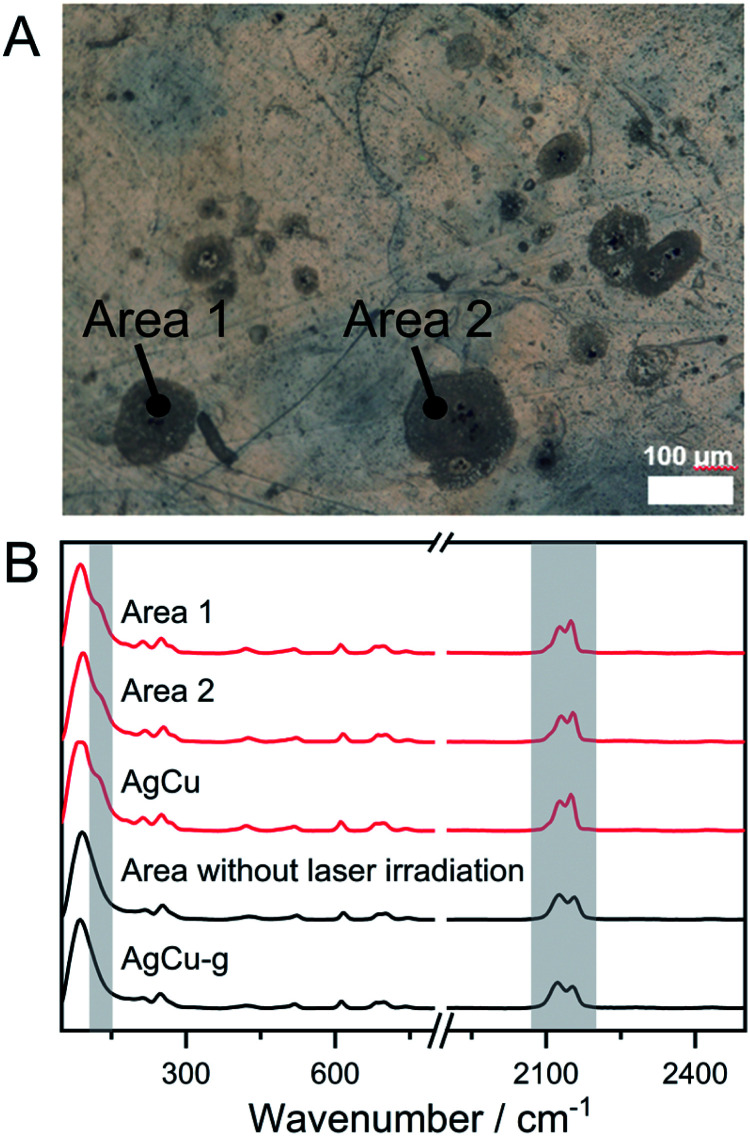
(A) Surface of the AgCu-g-CuSO_4_ monolith under the microscope. Areas 1 and 2 are irradiated using a 47 μm size laser. Other grey spots are also crystallized domains. (B) Confocal Raman spectra at areas 1 and 2, and the area without laser irradiation. Spectra of pure AgCu and AgCu-g are also shown.

The phase transition from amorphous to crystal by laser irradiation is a crucial phenomenon for the preparation of glass ceramics and phase memory devices.^55–58^ For instance, crystal and amorphous states of ternary alloy GeSbTe systems have different reflectance, and they are used in the Blu-ray disc. The phase transition from M^N^M^C^-g to M^N^M^C^ has been confirmed by DSC and PXRD, which inspired us to demonstrate the glass-to-crystal transformation triggered by laser irradiation. AgCu-g was chosen because its lower *T*_c_ (116 °C) than CuAu-g (*T*_c_ = 146 °C), which means less thermal energy is required to induce crystallization. We first tried to induce crystallization of AgCu-g by direct irradiation of the monolith with a 730 nm laser . It was not successful due to the low absorption of the 730 nm laser in the glass. We then mixed anhydrous CuSO_4_ (particle sizes are *ca.* 2 μm) into the glasses as the laser absorber. It is known that Cu^2+^ in CuSO_4_ shows a photothermal effect from forbidden d–d transitions.^59,60^ Homogeneous distribution of the mixed CuSO_4_ particles would generate heat to induce the crystallization of local domains in the glasses. 20 mg of CuSO_4_ was mixed with 180 mg of AgCu-g by ball-milling under an Ar atmosphere. The PXRD pattern of the mixed powder AgCu-g-CuSO_4_ contains characteristic peaks of only CuSO_4_ (Fig. S22A†). Reflectance diffusion spectra of AgCu-g-CuSO_4_ showed an absorption band at 700 nm corresponding to the absorption of CuSO_4_ (Fig. S22B†). The bulk monolith of AgCu-g-CuSO_4_ obtained by the hot-press process maintains its transparency (>60%, Fig. S21C†). CuSO_4_ particles with a diameter of 2 μm are homogeneously distributed on the surface of the monolith, confirmed by SEM and energy-dispersive X-ray spectroscopy (EDS) mapping (Fig. S22D and E†). Fig. 3A shows the example of the surface of the monolith of AgCu-g-CuSO_4_ observed using the microscope. We irradiated some spots on the monolith for 60 min using a 730 nm laser of 47 μm size. The irradiated domain turned grey. Confocal Raman spectroscopy within the range of 300 nm was carried out to identify the phase of irradiated areas (Fig. 3B). The Raman spectra of grey areas 1 and 2 in Fig. 3A showed an identical pattern to AgCu. The Ag–P vibration peak at 125.1 cm^−1^ was found in the Raman spectra. The relative intensities of the two cyanide peaks at 2130.8 and 2153.6 cm^−1^ are the same as AgCu. These results suggest that the crystalline AgCu phase was generated in areas 1 and 2 upon laser irradiation. The change was not observed in areas without laser irradiation, as also shown in Fig. 3B. This observation further proves the occurrence of glass-to-crystal transition through laser irradiation. Laser-induced crystallization was also observed on the surface of AgCu-g-CuSO_4_ powder pellets (Fig. S23†).

## Conclusions

We have explored three luminescent CP glasses M^N^M^C^-g composed of d^10^ metal cyanides and triphenylphosphine. Structural analyses revealed that the glasses are constructed from 1D chains which originate from their crystal structures. Higher structural dynamics in M^N^M^C^-g were confirmed by solid-state NMR. The disordered structures and enhanced molecular dynamics contributed to a shorter photoluminescence lifetime of M^N^M^C^-g than crystalline M^N^M^C^. Centimeter scale glass monoliths were prepared by the hot-press technique, and they exhibited both strong emission and high transparency over 80% from 400 to 2000 nm. Furthermore, the bulk transparent glass doped with a light absorber demonstrated the laser-induced crystallization at the target spot. The strong photoluminescence at room temperature and soft, moldable character of CP/MOF glasses in micro to macro scales will provide opportunities in lighting and photonic applications.

## Data availability

All data needed to evaluate the conclusions in the paper are present in the paper and supporting information. The data that support the findings of this study are available from the corresponding author upon request.

## Author contributions

S. H. designed the project. Z. F. and C. D. synthesized the compounds. Z. F. conducted and analyzed PXRD, TGA, DSC, SEM, FT-IR, Raman, diffusion reflectance, photoluminescence, EXAFS, XAS and laser irradiation experiments. A. D., R. Z. and S. T. conducted and analyzed photoluminescence lifetime. Y-S. W. conducted Rietveld analysis. S. H. and Z. F. wrote the paper.

## Conflicts of interest

There are no conflicts to declare.

## Supplementary Material

SC-013-D1SC06751F-s001

## References

[cit1] Horike S., Nagarkar S. S., Ogawa T., Kitagawa S. (2020). Angew. Chem., Int. Ed..

[cit2] Bennett T. D., Coudert F. X., James S. L., Cooper A. I. (2021). Nat. Mater..

[cit3] Fonseca J., Gong T., Jiao L., Jiang H.-L. (2021). J. Mater. Chem. A.

[cit4] Horike S., Ma N., Fan Z., Kosasang S., Smedskjaer M. M. (2021). Nano Lett..

[cit5] Chen W., Horike S., Umeyama D., Ogiwara N., Itakura T., Tassel C., Goto Y., Kageyama H., Kitagawa S. (2016). Angew. Chem., Int. Ed..

[cit6] Ogawa T., Takahashi K., Nagarkar S. S., Ohara K., Hong Y. L., Nishiyama Y., Horike S. (2020). Chem. Sci..

[cit7] Ma N., Kosasang S., Yoshida A., Horike S. (2021). Chem. Sci..

[cit8] Wang Y., Jin H., Ma Q., Mo K., Mao H., Feldhoff A., Cao X., Li Y., Pan F., Jiang Z. (2020). Angew. Chem., Int. Ed..

[cit9] Li J., Wang J., Li Q., Zhang M., Li J., Sun C., Yuan S., Feng X., Wang B. (2021). Angew. Chem., Int. Ed..

[cit10] Lin R., Li X., Krajnc A., Li Z., Li M., Wang W., Zhuang L., Smart S., Zhu Z., Appadoo D., Harmer J. R., Wang Z., Buzanich A. G., Beyer S., Wang L., Mali G., Bennett T. D., Chen V., Hou J. (2022). Angew. Chem., Int. Ed..

[cit11] Yu G., Lee C. H., Mihailovic D., Heeger A. J., Fincher C., Herron N., McCarron E. M. (1993). Phys. Rev. B Condens Matter..

[cit12] Zhao J., Zheng X., Schartner E. P., Ionescu P., Zhang R., Nguyen T. L., Jin D., Ebendorff-Heidepriem H. (2016). Adv. Opt. Mater..

[cit13] Meinardi F., Bruni F., Brovelli S. (2017). Nat. Rev. Mater..

[cit14] Meinardi F., Ehrenberg S., Dhamo L., Carulli F., Mauri M., Bruni F., Simonutti R., Kortshagen U., Brovelli S. (2017). Nat. Photonics.

[cit15] Qiao A., Tao H., Carson M. P., Aldrich S. W., Thirion L. M., Bennett T. D., Mauro J. C., Yue Y. (2019). Opt. Lett..

[cit16] Frentzel-Beyme L., Kloß M., Pallach R., Salamon S., Moldenhauer H., Landers J., Wende H., Debus J., Henke S. (2019). J. Mater. Chem. A.

[cit17] Ali M. A., Ren J., Zhao T., Liu X., Hua Y., Yue Y., Qiu J. (2019). ACS Omega.

[cit18] Vaidya S., Veselska O., Zhadan A., Diaz-Lopez M., Joly Y., Bordet P., Guillou N., Dujardin C., Ledoux G., Toche F., Chiriac R., Fateeva A., Horike S., Demessence A. (2020). Chem. Sci..

[cit19] Keggin J. F., Miles F. D. (1936). Nature.

[cit20] Hill J. A., Thompson A. L., Goodwin A. L. (2016). J. Am. Chem. Soc..

[cit21] Cliffe M. J., Keyzer E. N., Dunstan M. T., Ahmad S., De Volder M. F. L., Deschler F., Morris A. J., Grey C. P. (2019). Chem. Sci..

[cit22] Venkataraman D., Lee S., Moore J. S., Zhang P., Hirsch K. A., Gardner G. B., Covey A. C., Prentice C. L. (1996). Chem. Mater..

[cit23] Das C., Ogawa T., Horike S. (2020). Chem. Commun..

[cit24] Das C., Horike S. (2021). Faraday Discuss..

[cit25] Shaw B. K., Hughes A. R., Ducamp M., Moss S., Debnath A., Sapnik A. F., Thorne M. F., McHugh L. N., Pugliese A., Keeble D. S., Chater P., Bermudez-Garcia J. M., Moya X., Saha S. K., Keen D. A., Coudert F. X., Blanc F., Bennett T. D. (2021). Nat. Chem..

[cit26] Ghazzali M., Jaafar M. H., Akerboom S., Alsalme A., Al-Farhan K., Reedijk J. (2013). Inorg. Chem. Commun..

[cit27] Ghazzali M., Jaafar M. H., Al-Farhan K., Akerboom S., Reedijk J. (2012). Inorg. Chem. Commun..

[cit28] Jaafar M., Pevec A., Akerboom S., Alsalme A., Al-Farhan K., Ghazzali M., Reedijk J. (2014). Inorg. Chim. Acta.

[cit29] Ito K., Moynihan C. T., Angell C. A. (1999). Nature.

[cit30] Greaves G. N., Sen S. (2007). Adv. Phys..

[cit31] Qiao A., Bennett T. D., Tao H., Krajnc A., Mali G., Doherty C. M., Thornton A. W., Mauro J. C., Greaves G. N., Yue Y. (2018). Sci. Adv..

[cit32] Liu M., McGillicuddy R. D., Vuong H., Tao S., Slavney A. H., Gonzalez M. I., Billinge S. J. L., Mason J. A. (2021). J. Am. Chem. Soc..

[cit33] Ohara Y., Hinokimoto A., Chen W., Kitao T., Nishiyama Y., Hong Y. L., Kitagawa S., Horike S. (2018). Chem. Commun..

[cit34] Nascimento M. L. F., Souza L. A., Ferreira E. B., Zanotto E. D. (2005). J. Non-Cryst. Solids.

[cit35] Hadjiivanov K. I., Panayotov D. A., Mihaylov M. Y., Ivanova E. Z., Chakarova K. K., Andonova S. M., Drenchev N. L. (2021). Chem. Rev..

[cit36] Schilt A. A. (2002). Inorg. Chem..

[cit37] Shao M., Li M.-X., Wang Z.-X., He X., Zhang H.-H. (2017). Cryst. Growth Des..

[cit38] Yoshino H., Yamagami K., Wadati H., Yamagishi H., Setoyama H., Shimoda S., Mishima A., Le Ouay B., Ohtani R., Ohba M. (2021). Inorg. Chem..

[cit39] Deacon G. B., Green J. H. S. (1968). Spectrochim. Acta, Part A.

[cit40] Edwards D. A., Richards R. (1978). Spectrochim. Acta, Part A.

[cit41] Wang G., Ling Y., Wang H., Yang X., Wang C., Zhang J. Z., Li Y. (2012). Energy Environ. Sci..

[cit42] Cui H., Zhao W., Yang C., Yin H., Lin T., Shan Y., Xie Y., Gu H., Huang F. (2014). J. Mater. Chem. A.

[cit43] Li Y., Yan P., Guo C., Xu Q. (2020). Chem. Commun..

[cit44] Tomson N. C., Williams K. D., Dai X., Sproules S., DeBeer S., Warren T. H., Wieghardt K. (2015). Chem. Sci..

[cit45] Bowmaker G. A., Dyason J. C., Healy P. C., Engelhardt L. M., Pakawatchai C., White A. H. (1987). J. Chem. Soc., Dalton Trans..

[cit46] Nagarkar S. S., Kurasho H., Duong N. T., Nishiyama Y., Kitagawa S., Horike S. (2019). Chem. Commun..

[cit47] Araki H., Tsuge K., Sasaki Y., Ishizaka S., Kitamura N. (2005). Inorg. Chem..

[cit48] Kwon E., Kim J., Lee K. Y., Kim T. H. (2017). Inorg. Chem..

[cit49] Whittington C. L., Wojtas L., Larsen R. W. (2014). Inorg. Chem..

[cit50] Syzgantseva M. A., Stepanov N. F., Syzgantseva O. A. (2019). J. Phys. Chem. Lett..

[cit51] Florence J. M., Glaze F. W., Hahner C. H., Stair R. (1948). J. Am. Ceram. Soc..

[cit52] Rubin M. (1985). Sol. Energy Mater..

[cit53] Němec P., Olivier M., Baudet E., Kalendová A., Benda P., Nazabal V. (2014). Mater. Res. Bull..

[cit54] Ma L., Peng Y., Pei Y., Zeng J., Shen H., Cao J., Qiao Y., Wu Z. (2019). Sci. Rep..

[cit55] Stocker H. J. (1969). Appl. Phys. Lett..

[cit56] Yamada N., Ohno E., Akahira N., Nishiuchi K. i., Nagata K. i., Takao M. (1987). Jpn. J. Appl. Phys..

[cit57] Wong H. S. P., Raoux S., Kim S., Liang J., Reifenberg J. P., Rajendran B., Asheghi M., Goodson K. E. (2010). Proc. IEEE.

[cit58] Raoux S., Welnic W., Ielmini D. (2010). Chem. Rev..

[cit59] Honma T., Benino Y., Fujiwara T., Komatsu T. (2006). Appl. Phys. Lett..

[cit60] Komatsu T., Ihara R., Honma T., Benino Y., Sato R., Kim H. G., Fujiwara T. (2007). J. Am. Ceram. Soc..

