# Supplementary material for: Photoluminescent coordination polymer bulk glasses and laser-induced crystallization
Source: Chem Sci. 2022 Feb 24;13(11):3281–7. doi: 10.1039/d1sc06751f (PMC8926292; doi:10.1039/d1sc06751f)
Supplement: SC-013-D1SC06751F-s001 [file SC-013-D1SC06751F-s001.pdf]

Electronic Supplementary Material (ESI) for Chemical Science.
This journal is © The Royal Society of Chemistry 2022

Supporting Information for

Photoluminescent Coordination Polymer Bulk Glass and Laser Induced Crystallization

Zeyu Fan,[a] Chinmoy Das,[b] Aude Demessence,[c] Ruilin Zheng,[d] Setsuhisa Tanabe,[d] Yong-Sheng Wei,[b] Satoshi Horike[*abef]

[a] *Department of Synthetic Chemistry and Biological Chemistry, Graduate School of Engineering, Kyoto University, Katsura, Nishikyo-ku, Kyoto 615-8510, Japan*

[b] *AIST-Kyoto University Chemical Energy Materials Open Innovation Laboratory (ChEM-OIL), National Institute of Advanced Industrial Science and Technology (AIST), Yoshida-Honmachi, Sakyo-ku, Kyoto 606-8501, Japan*

[c] *Univ Lyon, Claude Bernard Lyon 1 University, UMR CNRS 5256, Institute of Researches on Catalysis and Environment of Lyon (IRCELYON), Villeurbanne, France*

[d] *Graduate School of Human and Environmental Studies, Kyoto University, Kyoto 606-8501, Japan*

[e] *Institute for Integrated Cell-Material Sciences, Institute for Advanced Study, Kyoto University, Yoshida-Honmachi, Sakyo-ku, Kyoto 606-8501, Japan*

[f] *Department of Materials Science and Engineering, School of Molecular Science and Engineering, Vidyasirimedhi Institute of Science and Technology, Rayong, 21210, Thailand*

*Email: horike@icems.kyoto-u.ac.jp (S.H)

Table of contents

Experimental section S2-S5
Supplementary figures and table S6-S20

Experimental

Materials

All chemicals were obtained from chemical companies and used without further purification. Gold(I) cyanide (AuCN), copper(I) cyanide (CuCN), silver(I) cyanide (AgCN), triphenylphosphine (PPh_3), hexane, and dichloromethane were obtained from FUJIFILM Wako Pure Chemical Corporation (Japan).

Synthesis of {[Ag(PPh$_3$)$_2$][Au(CN)$_2$]}$_n$ (AgAu)

AgAu was synthesized according to previous literature with a slight modification. AuCN (0.223 g, 1 mmol) was added to a dichloromethane (40 mL) containing PPh_3 (0.524 g, 2 mmol). The mixture was stirred for 30 min to obtain a clear solution. Then, AgCN (0.134 g, 1 mmol) was added to the solution and further stirred for 24 h. After filtration, hexane (30 mL) was added to the filtrate to obtain white crystalline powder. The powder collected by filtration was washed with hexane several times and then dried *in vacuo* at room temperature overnight. Yield: 0.620 g (70%). Elemental analysis calculated for $C_{38}H_{30}AgAuN_2P_2$ (**AgAu**): C, 51.73; H, 3.40; N, 3.18; experimental: C, 51.64; H, 3.40; N, 3.18.

Synthesis of {[Ag(PPh$_3$)$_2$][Cu(CN)$_2$]}$_n$·xH$_2$O (AgCu·xH$_2$O)

AgCu xH$_2$O was synthesized according to previous literature with a slight modification. AgCN (0.134 g, 1 mmol) was added to a CH_2Cl_2 (40 mL) containing PPh_3 (0.524 g, 2 mmol). The mixture was stirred for 30 min to obtain a clear solution. Then, CuCN (0.090 g, 1 mmol) was added to the solution with magnetically stirring for 24 h. After filtration, hexane (30 mL) was added to the filtrate to obtain white crystalline powder. The powder collected by filtration was washed with hexane several times and dried *in vacuo* at room temperature overnight. Yield: 0.685 g (92%).

Synthesis of {[Cu(PPh$_3$)$_2$][Au(CN)$_2$]}$_n$·xH$_2$O (CuAu·xH$_2$O)

CuAu xH$_2$O was synthesized according to previous literature with a slight modification. AuCN (0.223 g, 1 mmol) was added to a CH_2Cl_2 (45 mL) containing PPh_3 (0.524 g, 2 mmol). The mixture was stirred for 0.5 h to obtain a clear solution. Then, CuCN (0.090 g, 1 mmol) was added to the solution with magnetically stirring for 24 h to obtain white crystalline powder. The powder collected by filtration was washed with CH_2Cl_2 several times and dried *in vacuo* at room temperature overnight. Yield: 0.597 g (68%).

Degas of CuAu·xH$_2$O and AgCu·xH$_2$O

CuAu·xH$_2$O and **AgCu·xH$_2$O** were heat under vacuum overnight at 140 °C and 100 °C, respectively to obtain the guest free phase **CuAu** and **AgCu**. Elemental analysis calculated for $C_{38}H_{30}CuAuN_2P_2$ (**CuAu**): C, 54.47; H, 3.59; N, 3.35; experimental: C, 55.43; H, 3.60; N, 3.39. Elemental analysis calculated for $C_{38}H_{30}AgCuN_2P_2$ (**AgCu**): C, 61.02; H, 4.04; N, 3.74; experimental: C, 60.78; H, 4.05; N, 3.83.

Glass formation of AgAu, CuAu, and AgCu

120 mg of polycrystalline powder was transferred into a 20 mL agate vessel with ten agate balls (10 mm in diameter) under argon atmosphere. The sample was ball-milled at 400 rpm which involves 10 min run and 10 min pause for 48 cycles produced **AgAu-g**, **CuAu-g** and **AgCu-g**.

Preparation of bulk glass monolith

100 mg of **CuAu-g**/**AgCu-g** powder was first pressed into pellet by a pellet DIE with 13 mm diameter. The pellet was set between two pieces of metal support and this setup was then transferred into a vacuum hot-press machine. The sample was preheated at 100/105 °C in a vacuum for 30 min and then 60 kN pressure was added. The pressure was maintained for 60 min. Transparent monolith was obtained after pressure was released and the system cooled to room temperature.

Physical measurements

Powder X-ray diffraction (PXRD) patterns were recorded on a Rigaku MiniFlex 600 operated at 40 kV and 15 mA with Cu $K\alpha$ radiation (λ = 1.54 Å). Synchrotron PXRD was measured at SPring-8 at 25 °C (BL02B2, λ = 0.799617 Å). The synchrotron PXRD data were used for Rietveld refinement of **CuAu** and **AgCu**. The initial structures for Rietveld refinement were constructed from the reported crystal structure of **AgAu**. The refinement was carried out using EXPO2014 software.

Thermogravimetric and differential thermal analyses (TG/DTA) were carried out using a Rigaku Thermoplus 8120 for measurement under nitrogen atmospheres. A weighed sample in an aluminum pan was heated from 30 to 500 °C with heating rate of 10 °C min^{-1}. The samples in the pan were observed in real-time.

Differential scanning calorimetry (DSC) was measured with Hitachi High-Tech Science Corporation DSC7020. A weighed sample in an aluminum pan was heated or cooled with a rate of 10 °C min^{-1}.

Scanning electronic microscopy (SEM) observation was carried out by JEOL Model JSM-7001F4 SEM system operating at 15.0 kV. The samples were deposited on carbon tape and coated with osmium prior to the measurement.

Microscope images were taken by a wide-range zoom lens VH-Z100T.

Fourier-transform infrared spectroscopy (IR) measurements were carried out using JASCO FTIR-6700 under air between the wavelength of 500 and 3500 cm^{-1}.

Raman spectra were collected by using Raman spectra was collected at room temperature by using Horiba Jobin Yvon LabRAM HR800 with semiconductor laser at 488 nm.

Diffusion reflectance spectrum and transmittance of glass monolith were recorded with a Shimadzu UV-3600 spectrophotometer with an ISR-3100 integral sphere unit.

Photoluminescence spectra were recorded using a JASCO FP-6600DS spectrofluorometer at 25 °C.

Photoluminescence lifetime measurement was measured by Quantaurus-Tau-C11367, Hamamatsu Photonics at 25 °C. The excitation wavelength was set as 365 nm.

Extended X-ray Absorption Fine-Structure Spectroscopy were collected on beamline BL01B1 at SPring-8. X-ray absorption spectra in the energy region of the Cu K-edge were measured in transmission mode. Fourier transformation was k^3-weighted in the k range from 3.0 to 12.5 Å^{-1}. The data processing and coordination number fitting were performed with Athena and Artemis software, respectively. All samples were measured in air at 25 °C. The resulting k^3-weighted radial distribution function was fitted by a FEFF calculation using single crystal data of **CuAu·xH$_2$O**.

X-ray total scattering data of samples in a quartz glass capillary (ϕ = 2 mm) filled with argon were collected at 25 °C with four CdTe and two Ge detectors covering the Q range up to 25 Å^{-1} at the BL04B2 beamline (61.377 keV) at SPring-8. The incident beam was monochromated at λ = 0.2020 Å. $G(r)$ is obtained from the Fourier transformation of $S(Q)$ with a Lorch modification function through Igor Pro software.

^{31}P solid-state MAS NMR was performed on a JNM-ECZ600R (JEOL RESONANCE Inc.) solid-state NMR spectrometer at 14.1 T at 25 °C at 20 kHz (3.2 mm rotor).

Setup for laser irradiation

The setup for laser irradiation is shown as below. A concentrator was placed 7 cm in front of sample to ensure the highest laser power. The PhoxX® 730 nm diode laser was used for experiment.

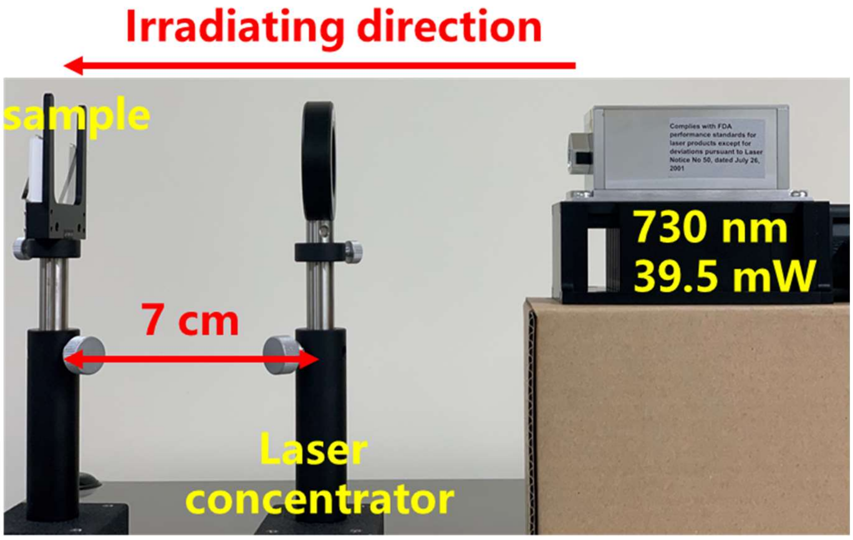

The focused beam size is calculated by using the following equation:

$$d = \frac{4\lambda M^2 f}{\pi D} = \frac{4 \times 730 \times 10^{-9} \times 1.05^2 \times 0.07}{\pi \times 1 \times 10^{-3}} \approx 47\ \mu\text{m}$$

Parameters:

d: beam size after concentration

D: beam size before concentration

f: focus distance of concentrator

λ: wavelength

M: quality of the beam

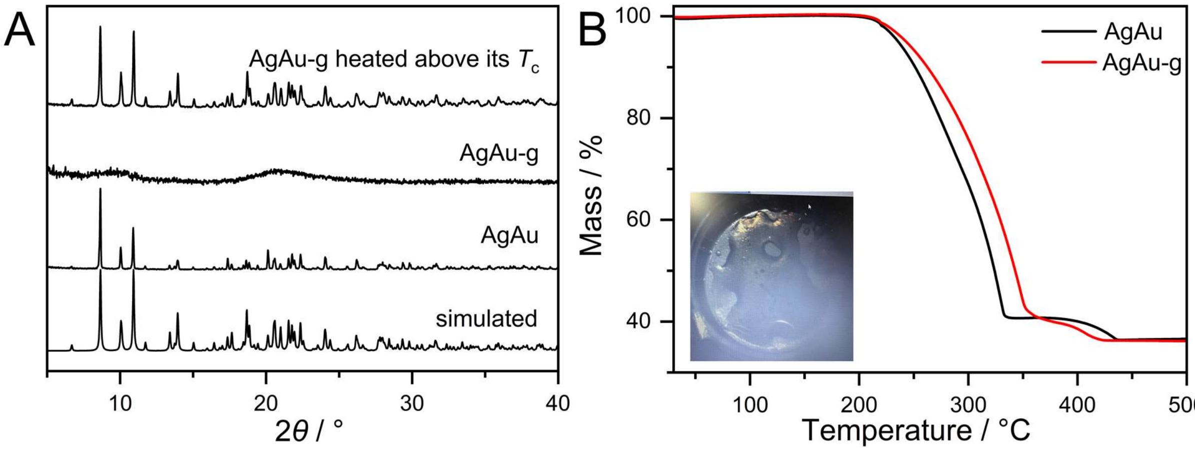

Fig. S1 (A) PXRD patterns of simulated **AgAu** from single crystal structure, synthesized **AgAu**, **AgAu-g**, and **AgAu-g** by heating above its T_c. (B) TGA profiles of **AgAu** and **AgAu-g** with a heating rate of 10 °C min^{-1} in N_2 atmosphere. Inset figure is the melt state of **AgAu** at T_m of 218 °C.

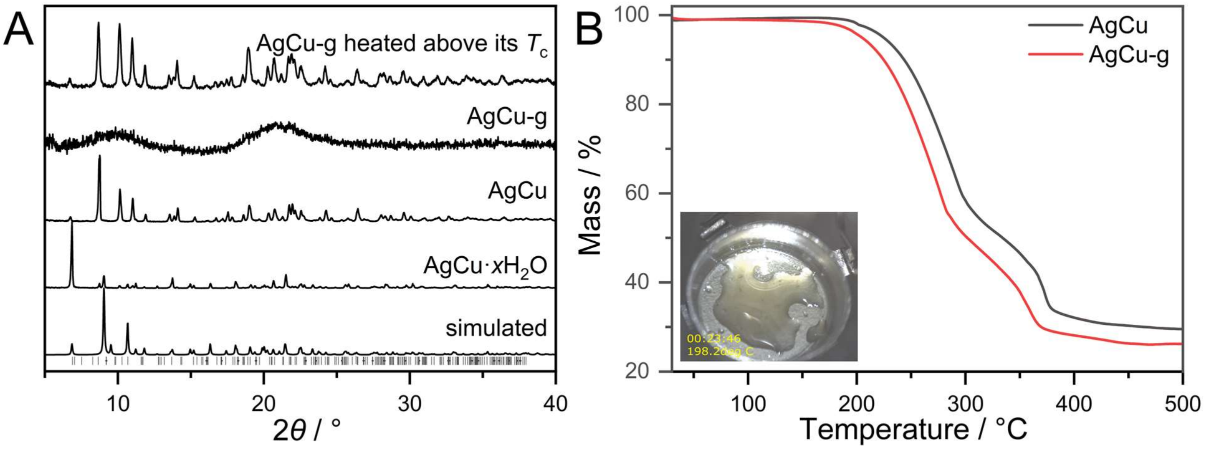

Fig. S2 (A) PXRD patterns of simulated **AgCu xH$_2$O** from the single crystal structure, synthesized **AgCu xH$_2$O**, **AgCu**, **AgCu-g**, and **AgCu-g** heated above its T_c. (B) TGA profile of **AgCu** and **AgCu-g** with a heating rate of 10 °C min^{-1} in N_2 atmosphere. Insert figure is melt state of **AgCu** at T_m of 197 °C.

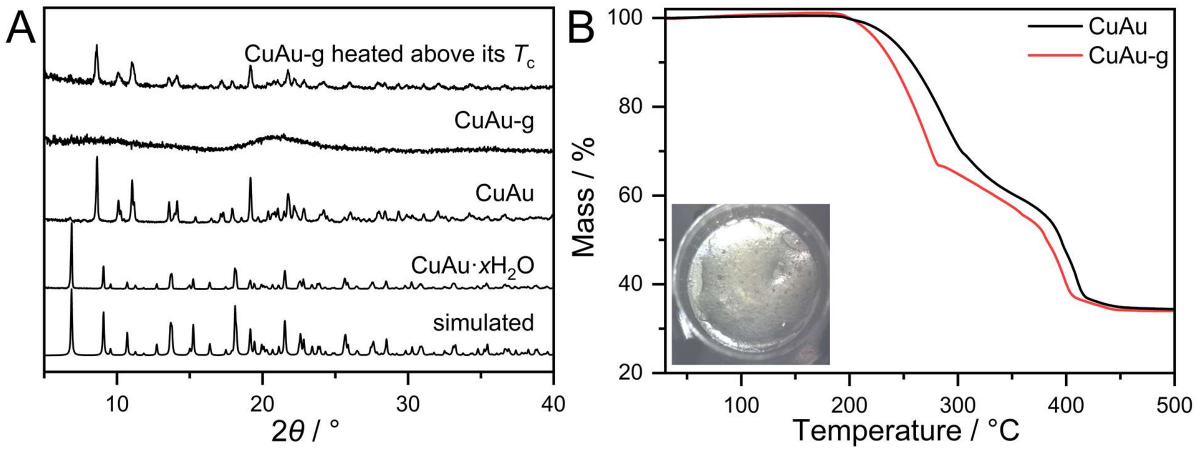

Fig. S3 (A) PXRD patterns of simulated **CuAu xH₂O** from the single crystal structure, synthesized **CuAu xH₂O**, **CuAu**, **CuAu-g**, and **CuAu-g** heated above its T_c. (B) TGA profile of **CuAu** and **CuAu-g** with a heating rate of 10 °C min^{-1} in N_2 atmosphere. Insert figure is melt state of **CuAu** at T_m of 198 °C.

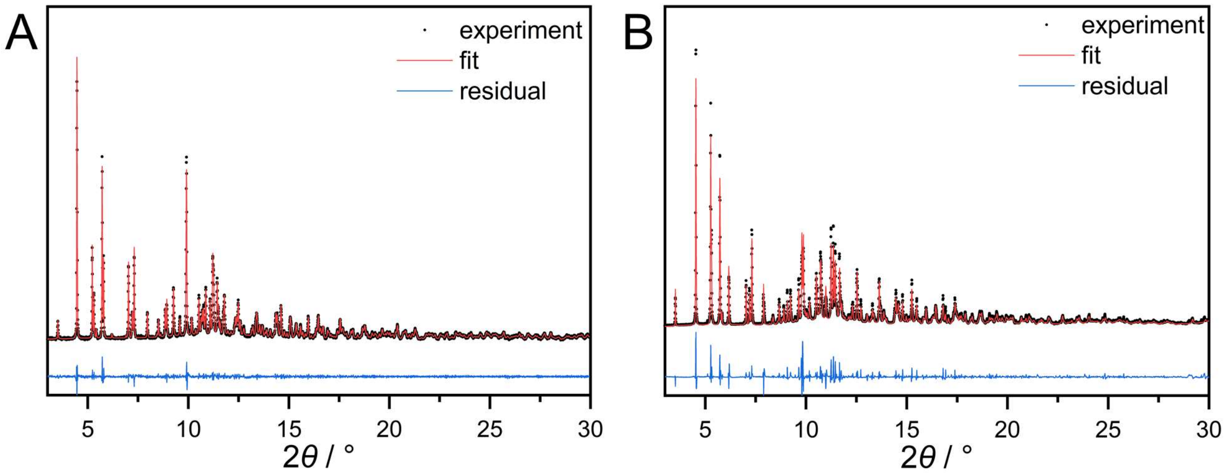

Fig. S4 (A) Result of Rietveld refinement of **CuAu**. Space group is P2$_1$/n. a = 9.56660 Å, b = 20.52415 Å, c = 16.99206 Å, β = 96.370°. R_p = 2.846%, R_{wp} = 3.703%. (B) Result of Rietveld refinement of **AgCu**. Space group is P2$_1$/n. a = 9.60682 Å, b = 20.23585 Å, c = 17.18036 Å, β = 95.931°. R_p = 5.523%, R_{wp} = 6.128%. λ = 0.799617 Å.

Table S1. Calculated ΔH_m and ΔS_m during melting of three ***M***

	T_m/℃	ΔH / kJ mol	ΔS / J mol K^{-1}
CuAu	198	50	105
AuAg	218	65	130
AgCu	197	53	114

Table S2. Elemental analysis of ***M*^{N}*M*^{C}s** before and after the melt-quench process.

	C / %	H / %	N / %
**AgAu** calculated	51.73	3.40	3.18
**AgAu** as-synthesized	51.64	3.40	3.18
**AgAu** after melt-quench	51.61	3.38	3.01
**CuAu** calculated	54.47	3.59	3.35
**CuAu** as-synthesized	55.43	3.60	3.39
**CuAu** after melt-quench	54.94	3.59	3.46
**AgCu** calculated	61.02	4.04	3.74
**AgCu** as-synthesized	60.78	4.05	3.83
**AgCu** after melt-quench	60.20	3.94	3.73

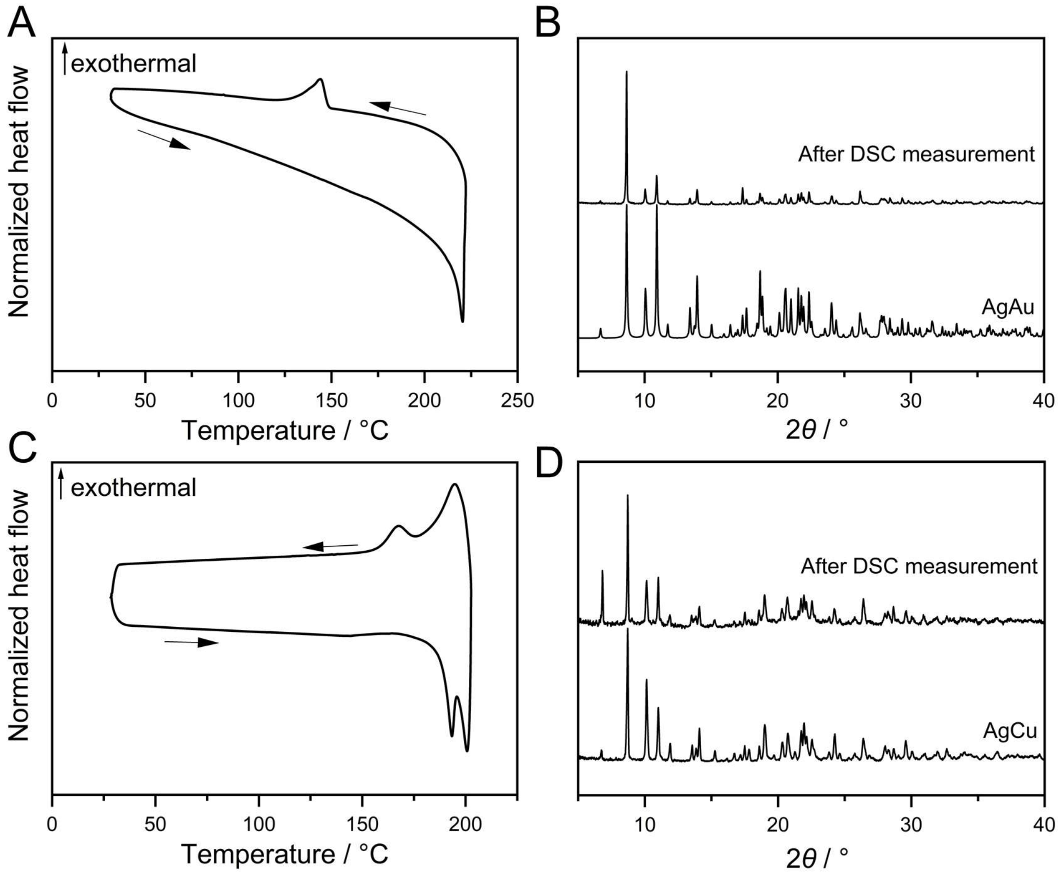

Fig. S5 (A) DSC profile of **AgAu**. (B) PXRD pattern of **AgAu** after DSC measurement. (C) DSC profile of **AgCu**. (D) PXRD pattern of **AgCu** after DSC measurement. DSC was measured under N_2 atmosphere with a heating rate of 10 °C min^{-1}.

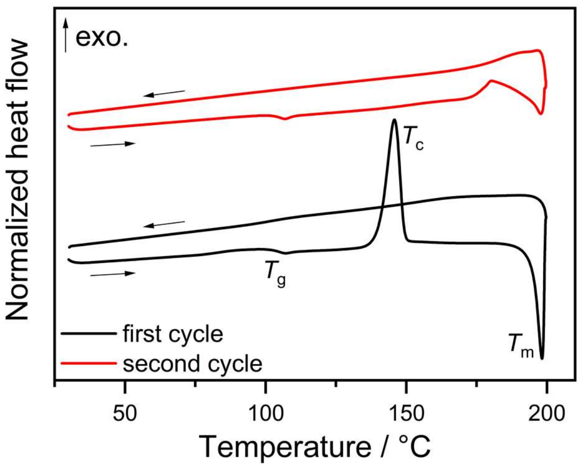

Fig. S6 DSC profile of ground **CuAu-MQG** with two heating-cooling cycles. DSC was measured under N_2 atmosphere with a heating rate of 10 °C min^{-1}.

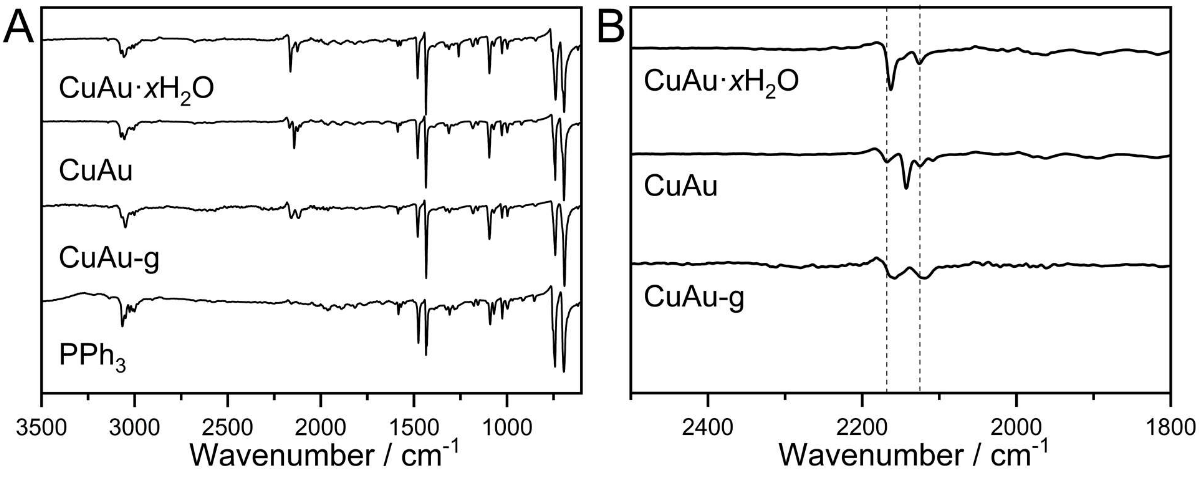

Fig. S7 (A) FT-IR spectra of **CuAu·*x*H$_2$O**, **CuAu**, **CuAu-g** and PPh$_3$. (B) Zoomed FT-IR spectra of of **CuAu·*x*H$_2$O**, **CuAu**, **CuAu-g**.

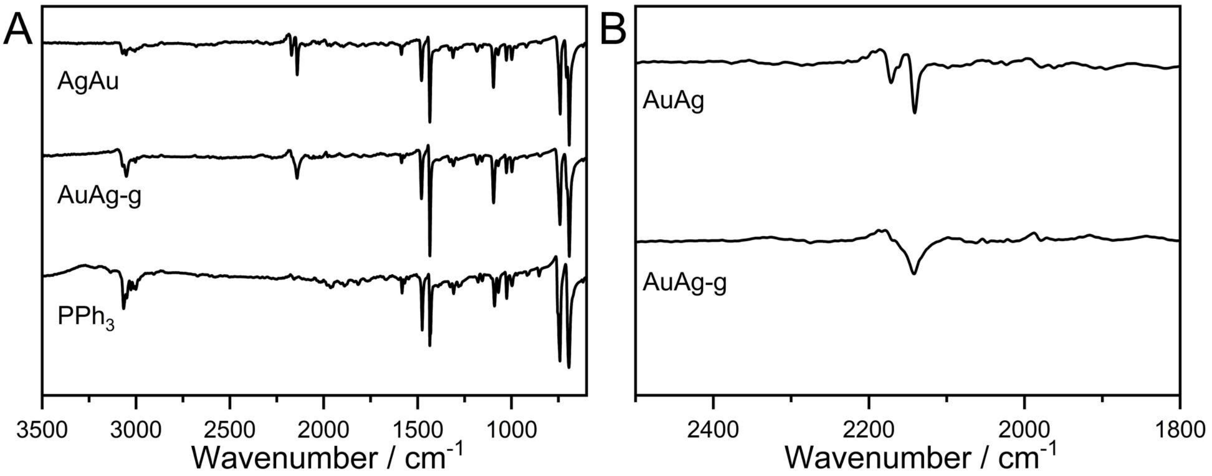

Fig. S8 (A) FT-IR spectra of **AgAu**, **AgAu-g**, and PPh$_3$. (B) Zoomed FT-IR spectra of **AgAu**, **AgAu-g**.

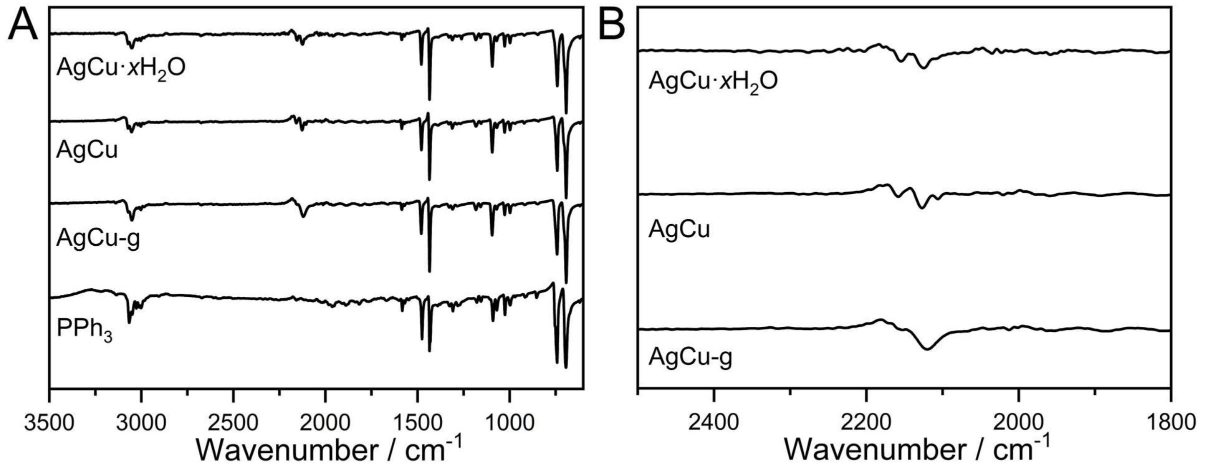

Fig. S9 (A) FT-IR spectra of **AgCu·*x*H$_2$O**, **AgCu**, **AgCu-g**, and PPh$_3$. (B) Zoomed FT-IR spectra of **AgCu·*x*H$_2$O**, **AgCu**, **AgCu-g**.

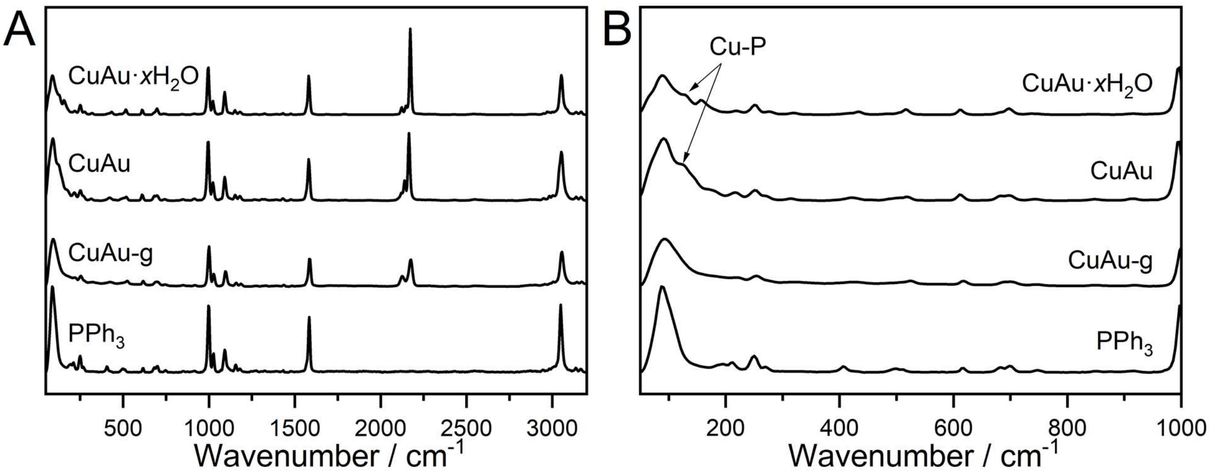

Fig. S10 Raman spectra of **CuAu·*x*H$_2$O**, **CuAu**, **CuAu-g** and PPh$_3$. (B) Zoomed Raman spectra of **CuAu·*x*H$_2$O**, **CuAu**, **CuAu-g** and PPh$_3$.

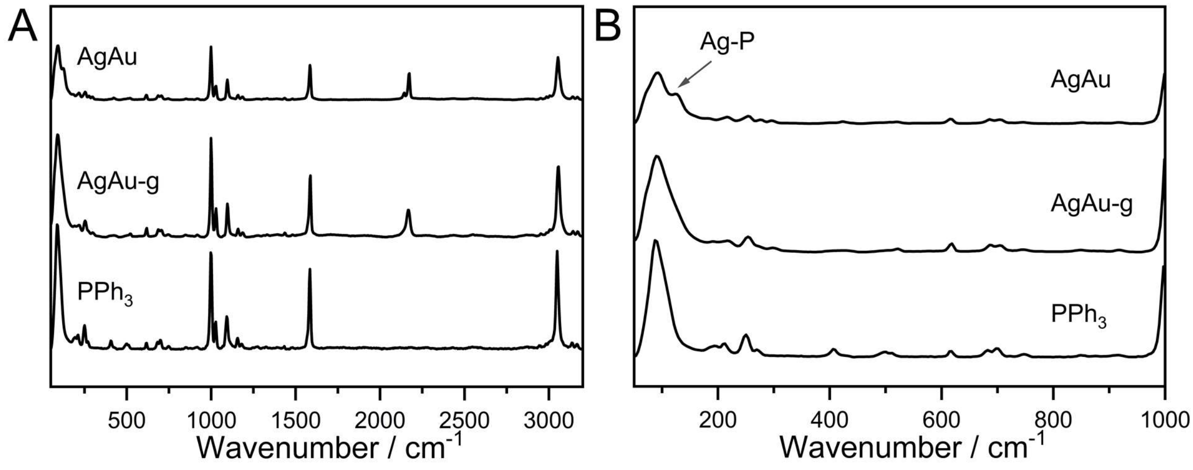

Fig. S11 (A) Raman spectra of **AgAu**, **AgAu-g**, and PPh_3. (B) Zoomed Raman spectra of **AgAu**, **AgAu-g**, and PPh_3.

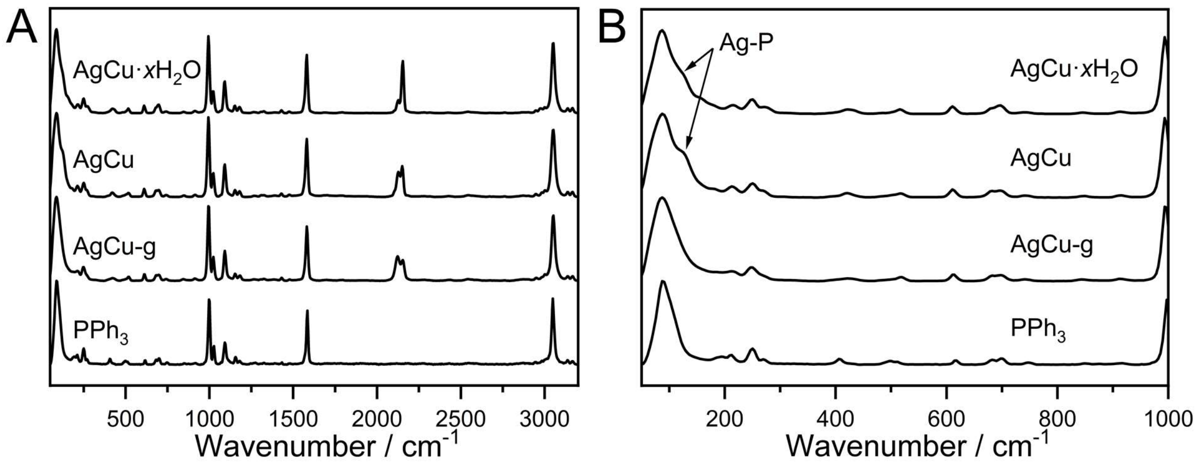

Fig. S12 (A) Raman spectra of **AgCu·*x*H₂O**, **AgCu**, **AgCu-g**, and PPh_3. (B) Zoomed Raman spectra of of **AgCu·*x*H₂O**, **AgCu**, **AgCu-g**, and PPh_3.

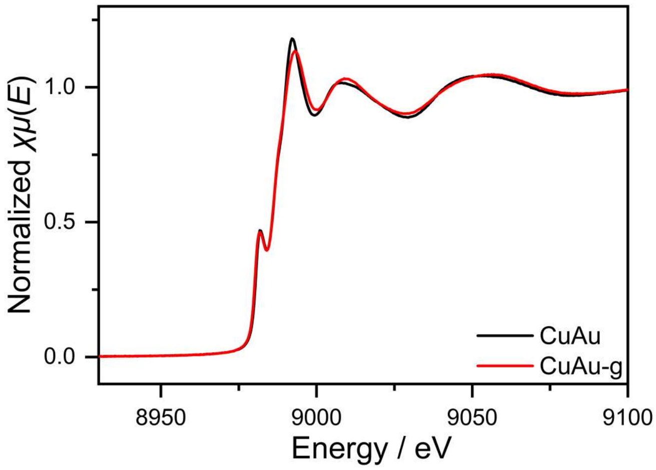

Fig. S13 Cu K-edge XANES spectra of **CuAu** and **CuAu-g**.

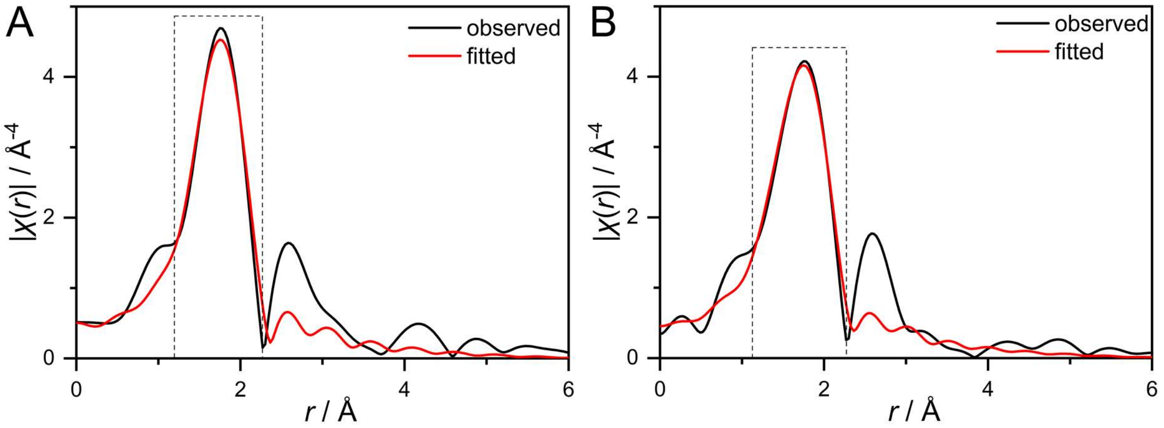

Fig. S14. k^3-weighted RDFs of (A) **CuAu** and (B) **CuAu-g**.

RDF fitting parameters for **CuAu** and **CuAu-g**.

Coordinating Atoms	*Coordination Number*	*R* / Å	S_0^2	σ^2	ΔE_0 / eV	*R-factor*
N	2.3 (±0.4)	1.98 (±0.02)	0.9	0.008	8.424	0.006
P	2.0 (±0.2)	2.29 (±0.01)	0.9	0.008	8.424	0.006

Coordinating Atoms	*Coordination Number*	*R* / Å	S_0^2	σ^2	ΔE_0 / eV	*R-factor*
N	2.2 (±0.8)	2.00 (±0.01)	0.9	0.009	8.081	0.007
P	2.0 (±0.2)	2.28 (±0.01)	0.9	0.009	8.081	0.007

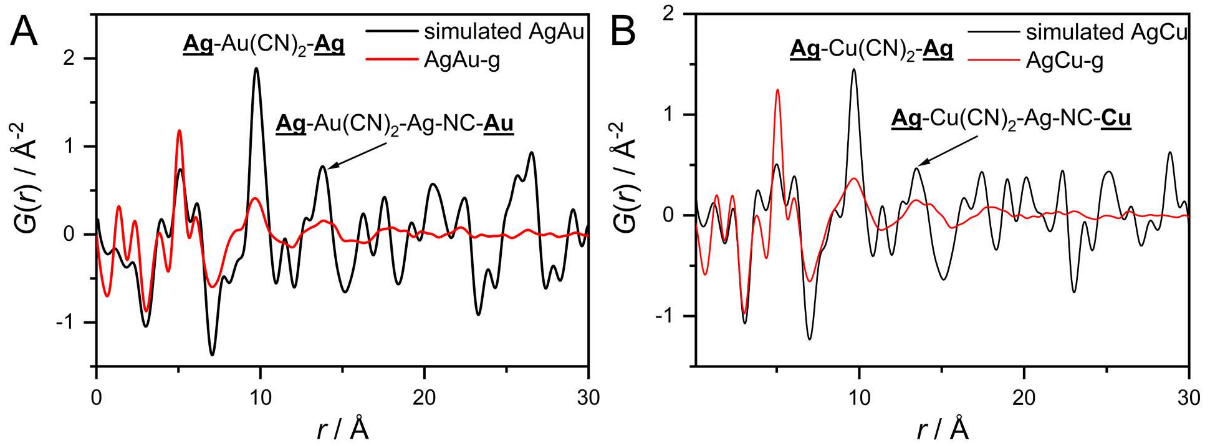

Fig. S15 PDF profiles of (A) **AuAg-g** and (B) **AgCu-g** compared with simulated PDF profiles of **AgAu** and **AgCu** from their crystal structures.

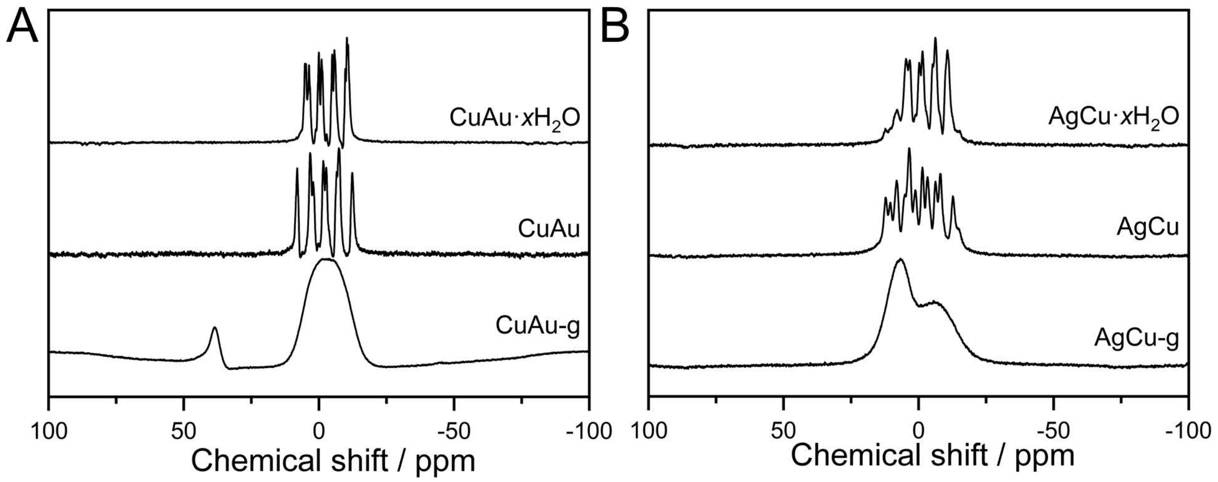

Fig. S16 Solid state ^{31}P MAS NMR of (A) **CuAu·xH$_2$O**, **CuAu**, **CuAu-g** and (B) **AgCu·xH$_2$O**, **AgCu** and **AgCu-g** at 25 °C.

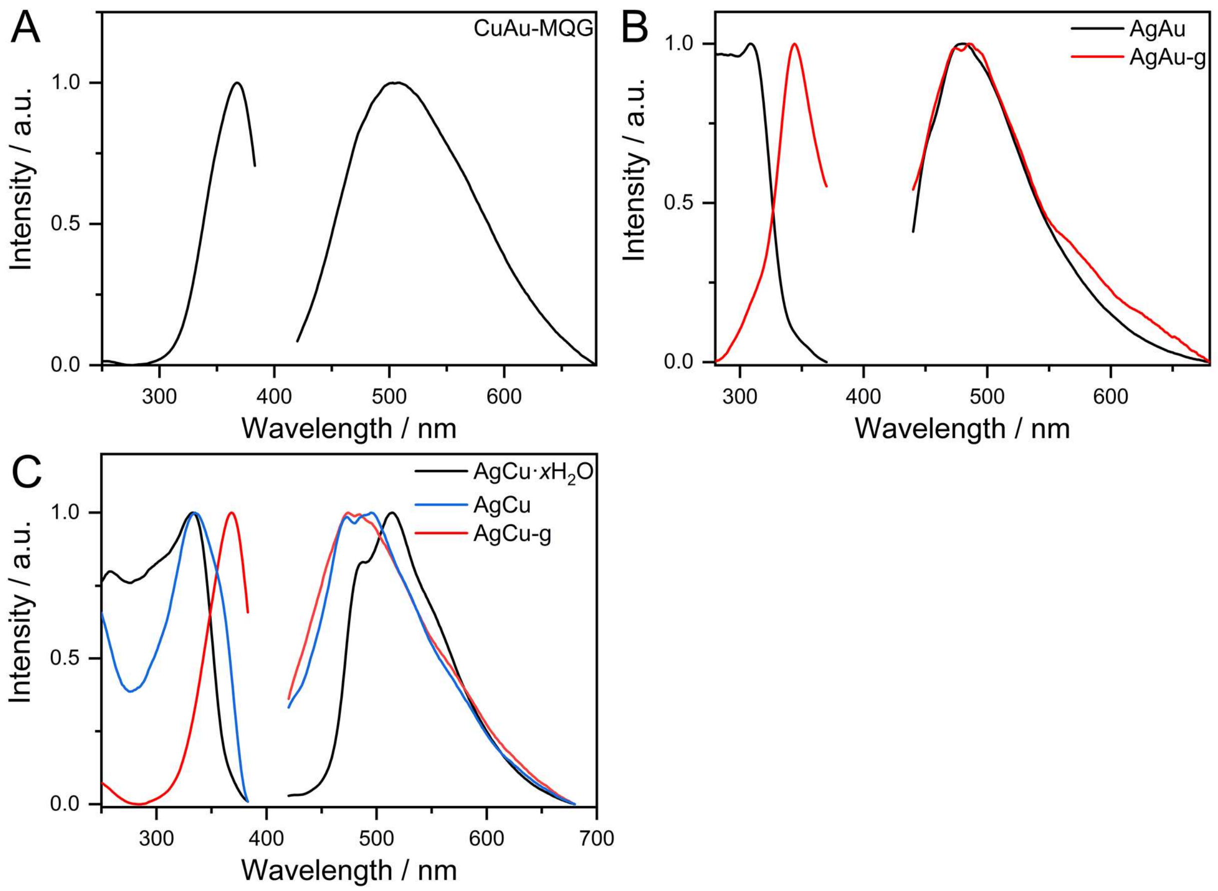

Fig. S17 Excitation and emission spectra of (A) **CuAu-MQG**, (B) **AgAu**, **AgAu-g** and (C) **AgCu·*x*H_2O**, **AgCu** and **AgCu-g** at 25 °C.

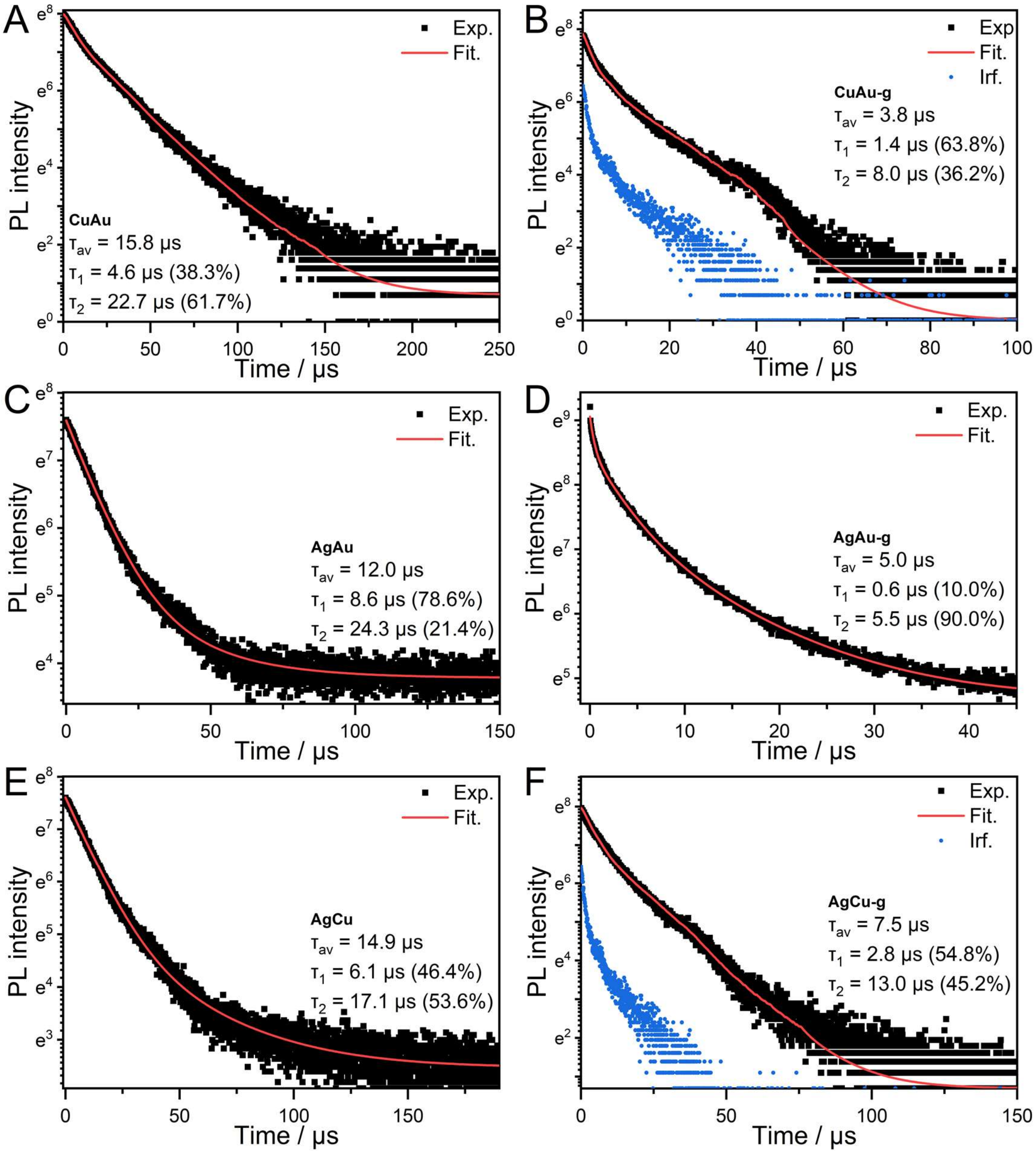

Fig. S18 Photoluminescent lifetime measurements of $\boldsymbol{M^{N}M^{C}}$ and $\boldsymbol{M^{N}M^{C}}$**-g** at 25 °C.

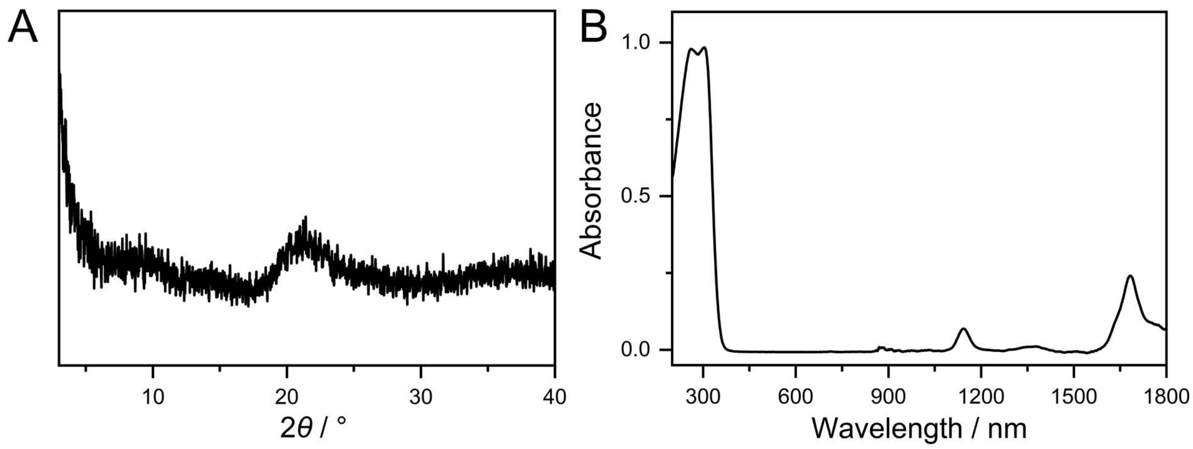

Fig. S19 (A) PXRD pattern of **CuAu-g** monolith made by hot-press. (B) Reflectance diffusion spectra of PPh_3.

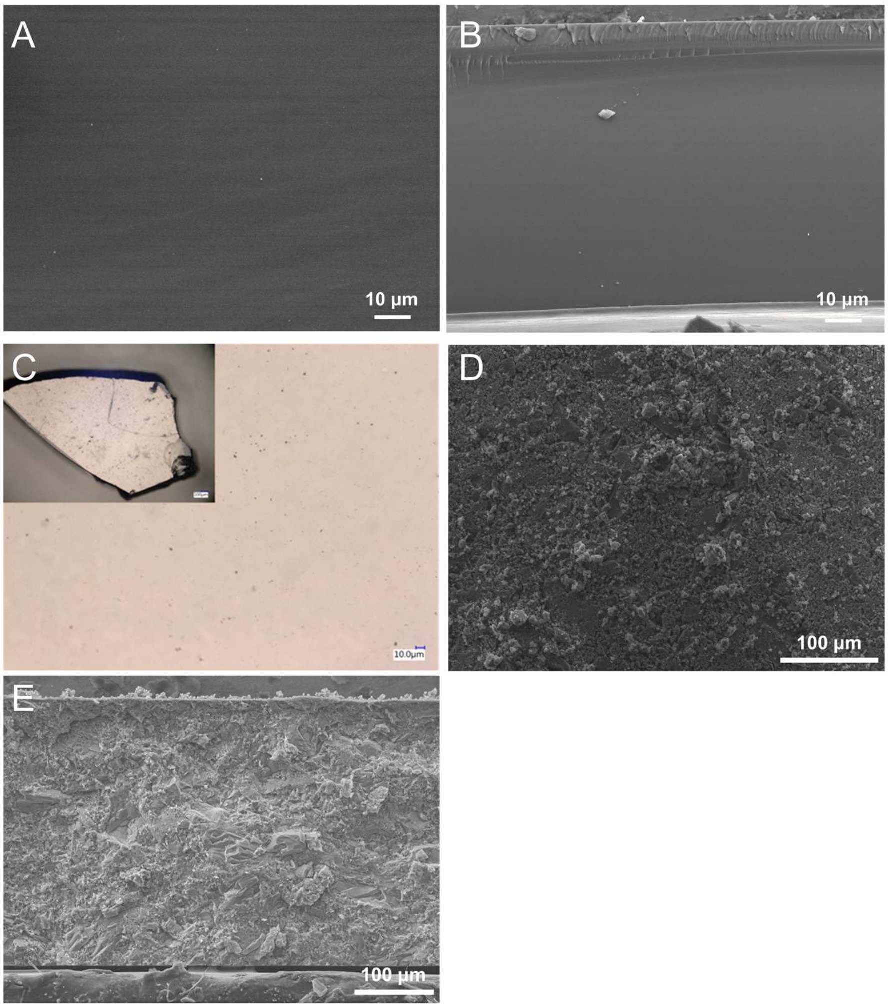

Fig. S20 (A) Surface and (B) cross-section SEM images of **CuAu-g** monolith. (C) Microscope image of **CuAu-g** monolith. (D) Surface and (E) cross-section SEM images of **CuAu** pellet.

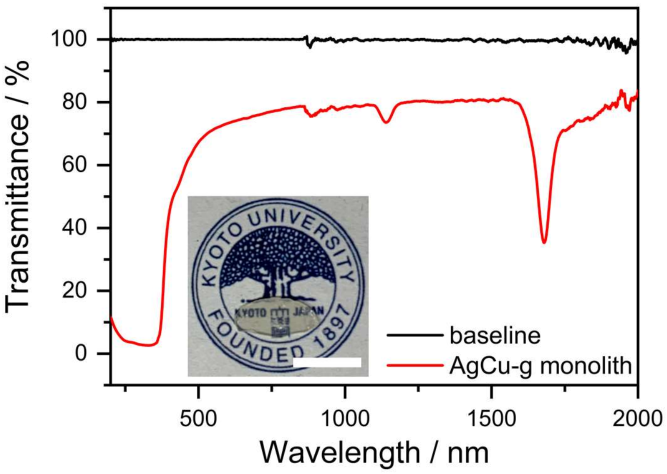

Fig. S21 Transmittance of **AgCu-g** monolith. Inset figure is the photo of **AgCu-g** monolith made by hot-press. The scale bar is 1 cm.

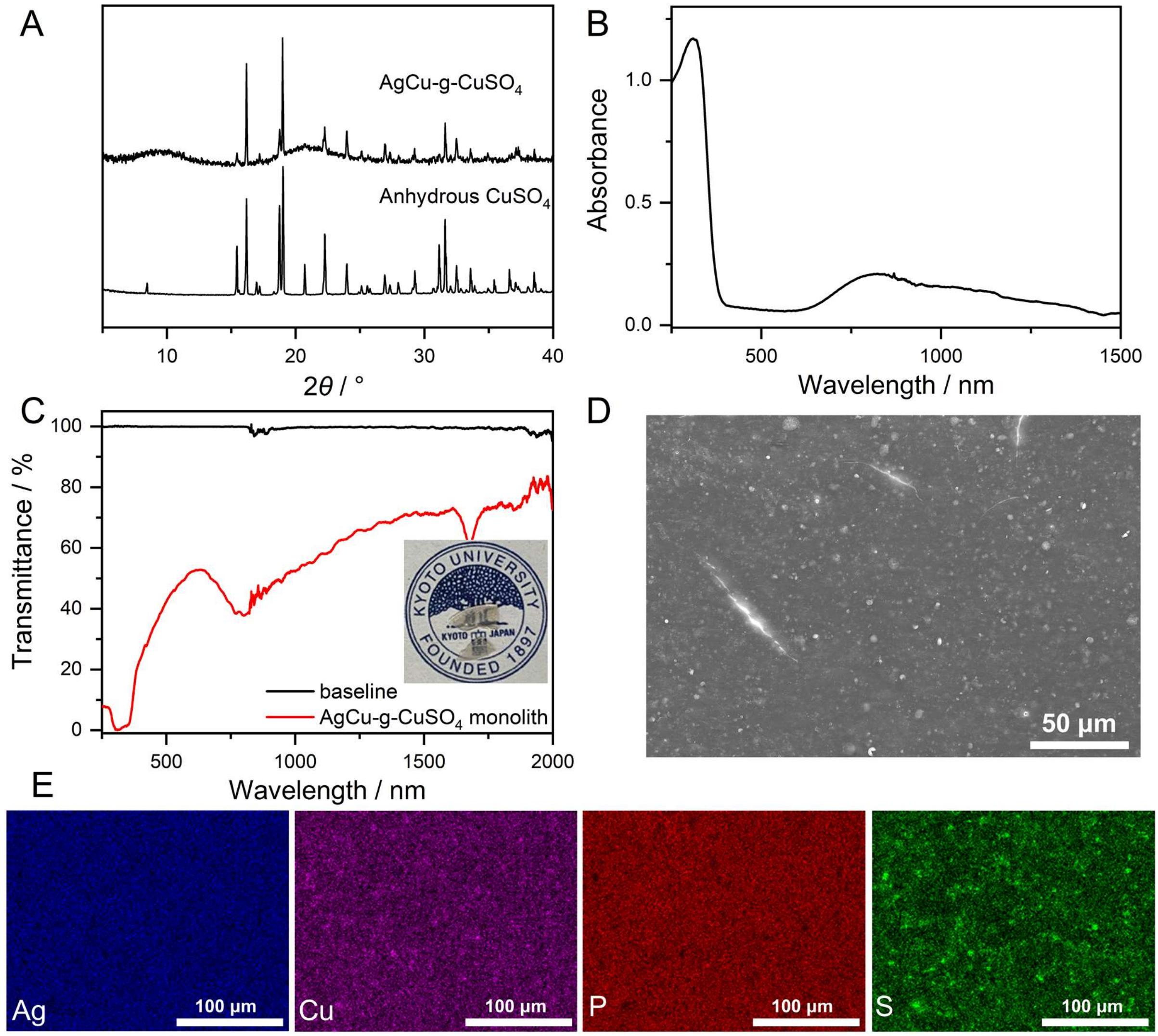

Fig. S22 (A) PXRD patterns of anhydrous $CuSO_4$ and **AgCu-g-$CuSO_4$**. (B) Reflectance diffusion spectra of **AgCu-g-$CuSO_4$**. (C) The transmittance of **AgCu-g-$CuSO_4$** monolith made by hot press. (D) SEM image and (E) EDS mapping on the **AgCu-g-$CuSO_4$** monolith.

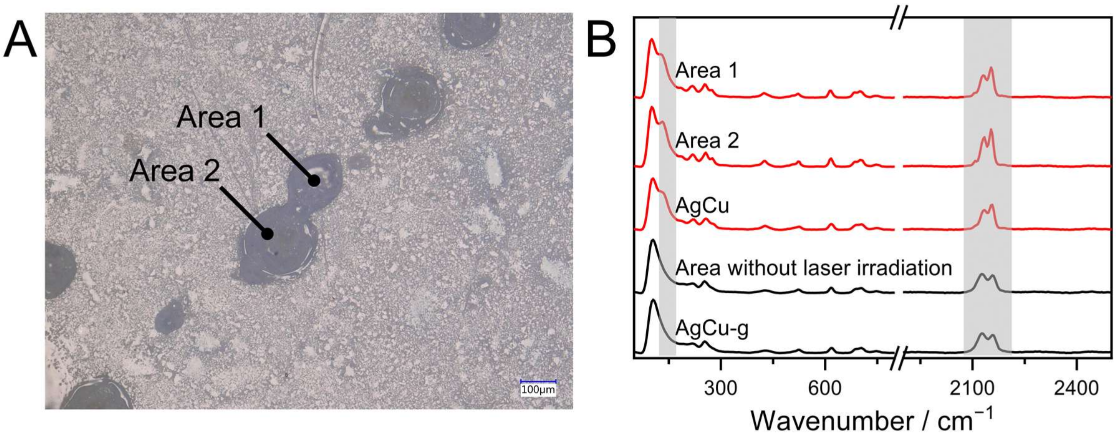

Fig. S23 (A) Surface of the **AgCu-g-CuSO₄** powder pellet under the microscope. Areas 1 and 2 are irradiated by 47 µm size of the laser. Other grey spots are also crystallized domain. (B) Confocal Raman spectra at areas 1 and 2, and the area without laser irradiation. Spectra of pure **AgCu** and **AgCu-g** are also shown.
